# Implementing the Risk Stratification and Clinical Management of Breast Cancer Families Using Polygenic Risk Score Evaluation: A Pilot Study

**DOI:** 10.3390/jpm14101034

**Published:** 2024-09-27

**Authors:** Barbara Rizzacasa, Vanessa Nicolì, Chantal Tancredi, Chiara Conte, Leila B. Salehi, Miriam Lucia Carriero, Giuliana Longo, Vincenzo Cirigliano, Luis Izquierdo Lopez, Bibiana Palao, Ilaria Portarena, Oreste Claudio Buonomo, Giuseppe Novelli, Michela Biancolella

**Affiliations:** 1 Department of Biomedicine and Prevention, University of Rome “Tor Vergata”, 00133 Rome, Italy; barbara.rizzacasa@uniroma2.it (B.R.); vanessa.nicoli.11@students.uniroma2.eu (V.N.); chantaltancredi@libero.it (C.T.); miriam.carriero@ptvonline.it (M.L.C.); novelli@med.uniroma2.it (G.N.); 2 Medical Genetics Unit, Tor Vergata University Hospital, 00133 Rome, Italy; chiaraconte@yahoo.it (C.C.); leilab.salehi@ptvonline.it (L.B.S.); 3 Veritas Intercontinental, 28020 Madrid, Spain; giuliana.longo@veritasint.com (G.L.); vin@veritasint.com (V.C.); luis@veritasint.com (L.I.L.); bibiana.palao@veritasint.com (B.P.); 4 Medical Oncology Unit, Tor Vergata University Hospital, 00133 Rome, Italy; ilaria.portarena@ptvonline.it; 5 Breast Unit, Department of Surgical Science, University of Rome “Tor Vergata”, 00133 Rome, Italy; o.buonomo@inwind.it; 6 Department of Biology, University of Rome “Tor Vergata”, 00133 Rome, Italy

**Keywords:** polygenic risk score, PRS, breast cancer, *BRCA1*, *BRCA2*, *PALB2*, *ATM*

## Abstract

Background: The identification of women at high risk of breast cancer (BC) is crucial for personalized screening strategies. Pathogenic and likely pathogenic variants (PVs/LPVs) in susceptibility risk genes explain part of the individual risk. Moreover, a polygenic background, summarized as a polygenic risk score (PRS), contributes to the risk of BC and may modify the individual risk in carrier and non-carrier members of BC families. Methods: We performed a retrospective pilot study evaluating PRS in women from a subset of high- (*BRCA1* and *BRCA2)* and moderate-risk (*PALB2* and *ATM*) BC families. We included PVs/LPVs carriers and non-carriers and evaluated a PRS based on 577,113 BC-associated variants. Using BOADICEA, we calculated the adjusted lifetime BC risk. Results: Our data showed that in *BRCA1/BRCA2* carriers, PVs have a major role in stratifying the lifetime risk, while PRS improves risk estimation in non-carriers of these families. A different scenario may be observed in *PALB2* and *ATM* families where PRS combined with PV/LPV carrier status gives a more informative lifetime risk. Conclusions: This study showed that in BC families, the PRS might help to quantify the weight of the genetic familial background, improving the individual risk stratification and contributing to personalized clinical management for carrier and non-carrier women.

## 1. Introduction

Breast cancer (BC) is the most common cancer in incidence and mortality among women [1]. It is well known that one of the major risk factors for BC is having a positive family history of the disease. Approximately 15–20% of all patients show familial clustering with a history of multiple breast or ovarian cancers (familial breast cancer, FBC); in these cases, a hereditary component is expected, but no obvious mutations are found to explain increased cancer rates. Conversely, 5–10% of cases are defined as hereditary and caused by germline pathogenic (PV) or likely pathogenic (LPV) variants in cancer predisposition genes [2,3,4,5,6,7]. While mutations in these genes are associated with a several-fold increased risk of developing hereditary breast and ovarian cancer (HBOC) [8], it has long been recognized that they have incomplete penetrance [9]. Notably, this incomplete penetrance could be partly explained by the polygenic background. Indeed, population-based genome-wide association studies (GWASs) led to the identification of numerous common low-risk cancer susceptibility variants [10,11,12,13]. When these variants are combined together and summarized into polygenic risk scores (PRSs), they can significantly weigh on the individual risk of cancer development [14,15,16,17,18]. The PRS can be used for a fine adjustment of the risk range of developing breast (and/or contralateral breast) cancer and ovarian cancer for women (both carriers and non-carriers of a PV/LPV) belonging to families with a positive history of BC. PRSs are calculated based on the cumulative effect of multiple genetic variants, which can provide a more nuanced understanding of an individual’s risk compared to traditional risk factors alone. Recent studies have demonstrated that individuals in the top decile of polygenic risk exhibit a 20.1% accumulated risk of breast cancer by age 70, indicating a substantial increase in lifetime risk compared to the general population [19]. Therefore, the involvement of such a polygenic background, besides the scientific implications about the pathophysiology of HBCO, has relevant clinical implications for genetic counseling and patient management. We performed a retrospective pilot study aimed at investigating the predictive power of PRS in a selected cohort of Italian women belonging to families with a positive history of BC, evaluating the PRS in both PV carrier and non-carrier members. We showed how the PRS might help quantify the weight of the genetic familial background, contributing not only to an individual risk stratification but also to personalized clinical management for both carrier and non-carrier women in BC families.

## 2. Materials and Methods

### 2.1. Patients’ Selection

This is a retrospective pilot study aimed to evaluate PRS in women belonging to BC families enrolled at Medical Genetics Complex Operative Unit, Policlinico Tor Vergata (Rome). The families selected for this study have been drawn starting from a vast cohort of women with breast cancer and strong family history after genetic counseling that underwent hereditary cancer testing with a 6 genes panel between January 2019 and January 2023. Next Generation Sequencing (NGS) analysis was performed by Ion Torrent S5 platform (ThermoFisher Scientific, Waltham, MA, USA) using a custom 6 genes panel (ThermoFisher Scientific) including *BRCA1, BRCA2, PALB2, ATM, CHEK2,* and *TP53.* The genetic variants identified by the NGS analysis have been classified using ClinGen gene-specific criteria (available at https://cspec.genome.network/cspec/ui/svi/, accessed on 26 September 2024) and American College of Medical Genetics and Genomics recommendations [20]. According to the clinical guidelines, relatives of PV/LPV carriers underwent segregation analysis using Sanger sequencing technique (SeqStudio Genetic Analyzers, Applied Biosystems, Waltham, MA, USA). We selected for the present pilot study 2 high-risk (*BRCA1* and *BRCA2*) and 2 moderate-risk (*PALB2* and *ATM*) BC families (Table 1). The variant identified in *PALB2* gene (Family 3 in Table 1) is not present in the reference databases, and it is classified as pathogenic (Class 5) according to ClinGen criteria specification for *PALB2* gene. All patients received and signed a written informed consent before peripheral blood collection. All the principles outlined in the Helsinki Declaration of 1975, as revised in 2013 [21], have been followed during the current study.

### 2.2. DNA Extraction and Whole Genome Sequencing

Total DNA was isolated from peripheral blood using the QIAGEN^®^ EZ1 DNA Blood 200 μL kit (Qiagen, Hilden, Germany) with the BioRobot EZ1 Workstation (Qiagen). The concentration and quality of DNA were determined using NanoDrop 1000 (Thermo Fisher Scientific) and the Qubit Fluorometer 2.0 (Thermo Fisher Scientific). Extracted DNA was processed with the Illumina^®^ DNA PCR-Free Prep Tagmentation library sample preparation kit and sequenced at low pass coverage on a NovaSeq 6000 System (Illumina, San Diego, CA, USA). The sequencing data were processed using dedicated software with customized imputation module to assess a common set of up to millions of genetic variants.

### 2.3. Polygenic Risk Score Assessment

For the evaluation of breast cancer PRS, we used the myHealthScore (https://www.veritasint.com/myhealthscore/, accessed on 26 September 2024) test performed in Veritas Intercontinental, which evaluates 577,113 genome-wide common genetic variants related to BC [22]. The PRS model is CE-IVD marked, designed, and validated by Allelica Inc exclusively for women (https://eu.allelica.com/prs/, accessed on 26 September 2024). Risk prediction is established based on the sum of the effect size of each SNP, weighted by the corresponding effect size from the PRS panel considering the ancestry-specific model. Allelica PRSs are calculated as a sum of risk effects of individual variants. The variance explained by the resulting score can theoretically reach the level of SNP heritability. The standard approach is to apply a PRS panel to a patient’s genotype data, sum these effects to generate a PRS value, ancestry adjust this value using PCs, and then compare it to an ancestry-matched reference population comprising individuals with known disease status to translate an individual’s PRS value into an estimate of their risk. Genetic ancestry is assessed by principal components of analysis (PCA) to adjust an individual’s PRS to his or her ancestry group. The PRS is finally aligned to ancestry-specific score distributions built using populations from a range of genetic ancestries. The result is considered at high risk when the PRS exceeds 2 times the odds ratio per standard deviation. A detailed description of this approach is reported in Busby et al., 2023 [23].

### 2.4. Evaluation of Adjusted Lifetime BC Risk

Polygenic risk score is an enhancing factor for breast cancer but must be interpreted in the context of the patient history and additional clinical information. To integrate all the factors impacting the lifetime risk of presenting breast cancer, CanRisk tool (https://www.canrisk.org/, accessed on 26 September 2024) [24] was used to assess the clinical risk. CanRisk is a web interface to BOADICEA, the Breast and Ovarian Analysis of Disease Incidence and Carrier Estimation Algorithm. This is one of the most important risk prediction models that allows the risk prediction in unaffected women considering multiple factors such as presence of cancer susceptibility mutations, polygenic risk score, family cancer history, personal clinical data, and lifestyle. At the same time, BOADICEA allows us to estimate the contralateral breast cancer (CBC) risk in previously affected women. Starting in 2013, BOADICEA model has been incorporated into National Institute for Health and Care Excellence guidelines for *BRCA1* and *BRCA2* mutation carrier risk estimation in the management of familial breast cancer [25]. The breast and ovarian cancer models used by CanRisk are described in Lee et al. 2019 [25] and Lee et al. 2022 [26].

## 3. Results

This pilot study is focused on the integration of predisposing genetic and environmental (smoke, diet, and physical activity habits as well as information about the use of oral contraceptives when available) BC factors in four selected BC families using the prediction model BOADICEA [25,26] in which the pedigree-based family history can be easily combined with the individual PRS value and with genetic test results with the aim of establishing a personal BC lifetime risk estimation. For each family, we performed the segregation analysis of the PV/LPV variant identified in the proband; then, we evaluated the PRS for each member and performed a BOADICEA analysis for the proband’s CBC lifetime risk and BC lifetime risk for unaffected family members.

### 3.1. Family 1

**Figure 1:**
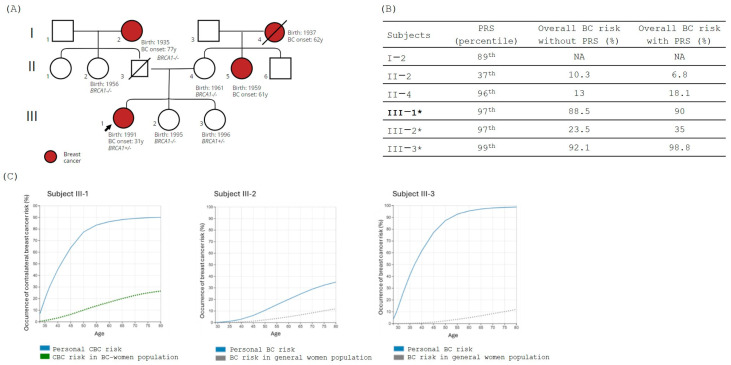
(**A**) Family tree; in red are shown the BC-affected individuals. The black arrow indicates the proband. Carrier status is mentioned in the figure, where +/− indicates the presence of the PV in heterozygous while −/− indicates the absence of the variant. (**B**) The table shows the polygenic risk score (PRS) obtained using myHealthScore test by Veritas Intercontinental and the evaluation of the overall BC risk calculated by age 80 without and with PRS using BOADICEA model. Family proband is reported in bold, and family members considered for the BOADICEA analysis are marked with “*”. NA (Not applicable) for subjects older than 80 years of age at the moment of the analysis. (**C**) Graphical representation of the percentage risk of developing BC (breast cancer) or CBC (contralateral breast cancer) during lifetime in *BRCA1* family members. The blue line corresponds to the individual personal risk. Green dot line indicates the estimated risk of CBC in BC affected women population. Gray dot line indicates the estimated risk of BC in the general population.

In Family 1, the segregation analysis for the pathogenic variant NM_007294.3:c.4117G>T, p.(Glu1373*) in the *BRCA1* gene identified in the proband (III-1) was performed for all family members enrolled in the study (*n* = 5) and the variant was identified in subject III-3 (Figure 1A).

Evaluation of PRS was performed for all the family members, and results, expressed in percentile, are reported in Figure 1B. For integration analysis in BOADICEA, we focused our attention on subjects III-1 (proband), III-2 (healthy, non-carrier), and III-3 (healthy, PV carrier) (Figure 1C). BOADICEA analysis for subject III-1 showed a very high risk of CBC during the lifetime, with a probability of 50% before age 45. Despite subjects III-1, III-2, and III-3 having a high PRS value (97th percentile of risk for III-1 and III-2, 99th percentile of risk for III-3), after integrating the family history and the personal clinical setting, we observed that the absence of the PV in s III-2 substantially decreases the BC lifetime risk compared to the carrier subjects III-1 and III-3 (Figure 1C) even though her personal lifetime risk remains remarkably higher than the general population, with a 35% risk of BC by age 80.

### 3.2. Family 2

**Figure 2:**
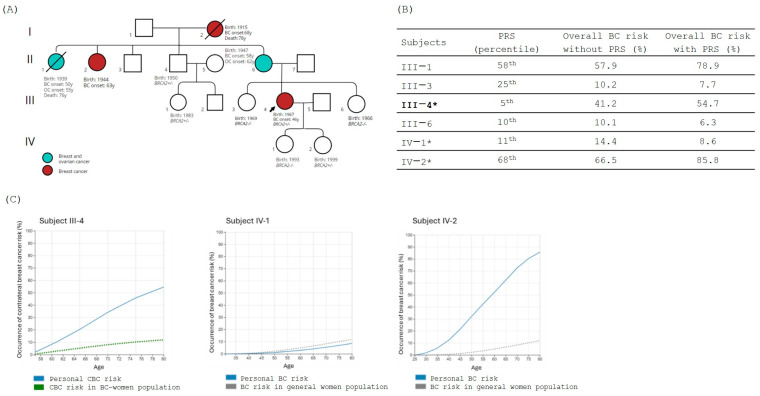
(**A**) Family tree; in red are shown the BC-affected individuals, while in light blue are shown subjects affected by both breast and ovarian cancers. The black arrow indicates the proband. Carrier status is mentioned in the figure, where +/− indicates the presence of the PV in heterozygous while −/− indicates the absence of the variant. (**B**) The table shows the polygenic risk score (PRS) obtained using myHealthScore test by Veritas Intercontinental and the evaluation of the overall BC risk calculated without and with PRS using BOADICEA model. Family proband is reported in bold, and subjects considered for the BOADICEA analysis are marked with “*”. (**C**) Graphical representation of the percentage risk of developing BC (breast cancer) or CBC (contralateral breast cancer) during lifetime in *BRCA2* family members. The blue line corresponds to the individual personal risk. Green dot line indicates the estimated risk of CBC in BC affected women population. Gray dot line indicates the estimated risk of BC in the general population.

In Family 2, members enrolled in the study (*n* = 5) underwent segregation analysis for the PV variant NM_000059.4:c.7680dup, p.(Gln2561Serfs*5) in the *BRCA2* gene identified in the proband (III-4). The variant was identified in subjects III-1 (proband’s 1st-grade cousin on the maternal side) and IV-2 (proband’s daughter) (Figure 2A), both in healthy status. PRS was evaluated for all the family members, and results, expressed in percentile, are reported in Figure 2B.

Among all the family members tested for the PRS, we selected the subject III-4 (proband, affected by BC and PV carrier) and her two daughters, subjects IV-1 (healthy, non-carrier) and IV-2 (healthy, PV carrier) for the integration analysis in BOADICEA. For proband III-4, diagnosed with BC at 46 years of age, the analysis showed a risk of CBC of about 55% by the age of 80 (Figure 2C). For subject IV-1, the integration in BOADICEA of all personal and clinical information combined with PRS led to the observation that her personal lifetime risk of BC is notably low, despite the positive family history, and comparable to the general population (Figure 2C). On the other hand, the carrier status of subject IV-2 combined with the positive family history and a higher value of PRS (68th percentile of risk) significantly increased her lifetime risk of BC, with a 20% chance of developing cancer by age 40 and more than 80% by age 80 (Figure 2C).

### 3.3. Family 3

**Figure 3:**
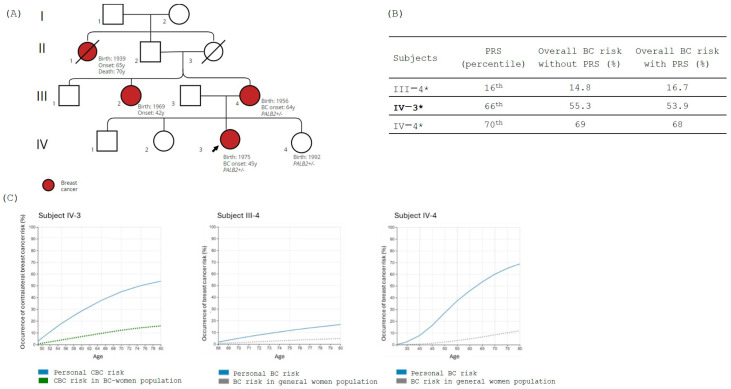
(**A**) Family tree; in red are the BC-affected individuals. The black arrow indicates the family proband. Carrier status is mentioned in the figure, where +/− indicates the presence of the PV in heterozygous while −/− indicates the absence of the variant. (**B**) The table shows the polygenic risk score (PRS) obtained using myHealthScore test by Veritas Intercontinental and the evaluation of the overall BC risk calculated without and with PRS using BOADICEA model. Family proband is reported in bold, and subjects considered for the BOADICEA analysis are marked with “*”. (**C**) Graphical representation of the percentage risk of developing BC (breast cancer) or CBC (contralateral breast cancer) during lifetime in *PALB2* family members. The blue line corresponds to the individual personal risk. Green dot line indicates the estimated risk of CBC in BC affected women population. Gray dot line indicates the estimated risk of BC in the general population.

Family 3 was screened for the pathogenic variant NM_024675.4:c.2351_2352delinsT, p.(Lys784IleFs*67) in the *PALB2* gene identified in the proband IV-3. Subjects III-4 (proband’s mother) and IV-4 (proband’s younger sister) were carriers (Figure 3A).

As shown in the family tree, the proband (IV-3) and III-4 are both affected by BC diagnosed at the age of 42 and 64, respectively, while the subject IV-4 is unaffected. PRS was evaluated for all the family members, and results, expressed in percentile, are reported in Figure 3B. Considering the CBC risk, BOADICEA analysis showed that subject IV-3 (the proband) has over 40% likelihood of CBC by the age of 70, while subject III- 4 shows 5% CBC risk by the same age. For subject IV-4, who is 31 years old and in good health condition, the integration of all risk factors (carrier status, PRS, and family history) in BOADICEA showed a 70% risk of developing BC by age 80 (Figure 3C).

### 3.4. Family 4

**Figure 4:**
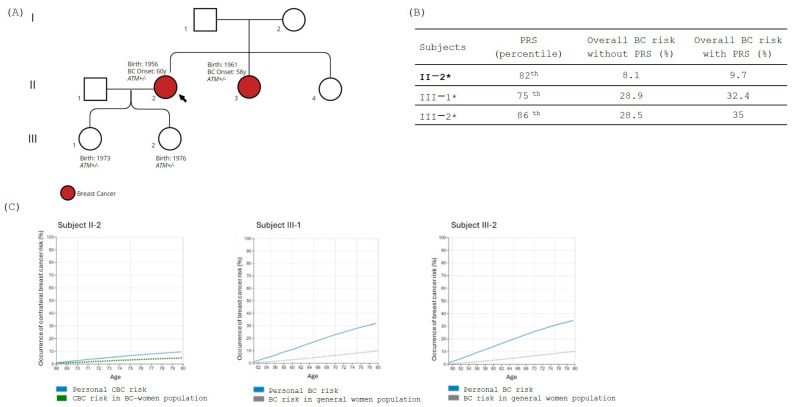
(**A**) Family tree; in red are the BC-affected individuals. The black arrow indicates family proband. Carrier status is mentioned in the figure, where +/− indicates the presence of the PV in heterozygous while −/− indicates the absence of the variant. (**B**) The table shows the polygenic risk score (PRS) obtained using myHealthScore test by Veritas Intercontinental and the evaluation of the overall BC risk calculated without and with PRS using BOADICEA model. Family proband is reported in bold, and subjects considered for the BOADICE analysis are marked with “*”. (**C**) Graphical representation of the percentage risk of developing BC (breast cancer) or CBC (contralateral breast cancer) during lifetime in *ATM* family members. The blue line corresponds to the individual personal risk. Green dot line indicates the estimated risk of CBC in BC affected women population. Gray dot line indicates the estimated risk of BC in the general population.

In Family 4, the LPV variant NM_000051.4:c.7515+1G>C, p.? in the *ATM* gene was identified in II-2 (proband), and subsequently also in III-1 and III-2, the two proband’s daughter, who are both in good health condition (Figure 4A).

We evaluated the PRS for all subjects, and it resulted in a high range of percentile of risk (76th–86th percentile), as shown in Figure 1B. BOADICEA analysis for the proband (II-2) showed ~10% of the risk of CBC at age 80 years despite the moderately high PRS value (Figure 4B,C). On the other hand, for both subjects III-1 and III-2, integration of carrier status, PRS, and family history using the BOADICEA model defined a lifetime risk of BC greater than 30% by age 80 (Figure 4C).

## 4. Discussion

Previous works have already shown the powerful potential of including a well-validated PRS for BC in clinical genetic services for the management of high-risk BC families [27,28]. Polygenic score assessment is independent of the risk associated with rare pathogenic variants related to breast cancer susceptibility genes, and there is growing evidence about the potential benefits of combining these two types of assessments [29] to improve risk prediction in different clinical situations. Lakeman and colleagues showed that the PRS evaluation for individuals belonging to BC families leads to substantially different patient stratification compared to the current risk prediction, which is primarily based on family history and status of PV carriers and non-carriers [27,28]. It is well known that pathogenic mutations in BC susceptibility genes do not show a complete penetrance, and this is true for both high- and moderate-risk genes. In this regard, Kuchenbacker et al. demonstrated a wide variation in the absolute risk of developing breast cancer for *BRCA1*/*BRCA2* mutation carrier women in the low and high percentile of risk determined by applying PRS [30]. Another study evaluating PV carriers in moderate-risk genes showed that using PRS, it is possible to reclassify more than 30% of *CHEK2* and around 50% of *ATM* PV carriers that have a lower lifetime BC risk compared to the one expected only considering the carrier status [31]. Moreover, Busby et al. state that PRS makes it possible to identify a significant fraction of the general population with a disease risk comparable to the risk of PV carriers in moderate-risk genes [22]. On a clinical level, it becomes clear that assessing the role of the genetic background might represent a very important aspect of defining a more precise personal risk, leading to appropriate screening and preventive measures both in BC families and the general population. In this study, we applied a CE-IVD marked PRS to evaluate its added value for the clinical management of BC families. This PRS model assesses 577,113 BC-associated variants, performing an ancestry-adjusted calculation of PRS [32]. We strongly believe that this aspect is a real added value to our analysis because the mean PRS varies by country (even among European populations) in accordance with the frequency of each variant included in the model, thus representing a remarkable limitation for general PRS applicability.

Moreover, as described in Busby et al., this PRS (Allelica 577k) has been shown to have an increased predictive performance compared with one of the most commonly used PRS models based on the evaluation of 313 genetic variants associated with BC [33] on the same PRS Testing dataset (statistic description is reported in Table 2) [22].

Regarding *BRCA1* and *BRCA2* families, our analysis is in line with the previous literature showing that PVs have a major role in the risk stratification of BC, and the polygenic background marginally influences the final overall risk score [34]. However, in non-carrier members of these families, the evaluation of the PRS might be a useful tool for a more concrete determination of the genetic background that can somehow balance the risk associated with a positive family history. Different considerations might be made in the BC families that segregate a pathogenic variant in a moderate-risk gene, such as *PALB2* and *ATM*. In this case, the different genetic backgrounds, summarized as PRS, could be involved in the different age onset of the disease, as we observed in the mutation carriers of the *PALB2* family (family 3) in which the PRS assessment better defines the individual lifetime BC risk compared to the one estimate only considering the PV carrier status [35], supporting the important role of the integration of PRS evaluation in patients’ clinical management. Similarly, in the *ATM* family (family 4), the integration in the BOADICEA model of PRS and family history results is useful for a better lifetime BC risk prediction for the two mutation carriers (III-1 and III-2) [35]. This pilot study showed that in BC families, the PRS might help to concretely quantify the weight of the genetic familial background contributing to an individual risk stratification that might lead to personalized clinical management for both PV carrier and non-carrier women in BC families. The implications of these findings are profound for clinical management. For women identified as high-risk through PRS, risk-reducing strategies such as prophylactic mastectomy or enhanced surveillance may be warranted [36]. Additionally, the dynamic nature of family history—where risk can change with new diagnoses in relatives—underscores the need for continuous risk assessment as part of a comprehensive management plan [37]. The clinical utility of the PRS in the cases analyzed in this study was important in family counseling, significantly influencing the estimation of cancer risk and concretely activating personalized clinical management strategies. For example, women identified with an elevated PRS were recommended more frequent screening to mitigate their elevated risk.

We are aware that our pilot study has several limitations. First, the number of families analyzed in this work is very small compared to other published papers [27,28], and this aspect did not allow us to perform statistical analysis of the data. Furthermore, not all subjects eligible for the segregation analysis were available, and ultimately, we had to exclude from our cohort all the male relatives since the PRS model used is CE-IVD marked only for women, further reducing the numbers of family members helpful for the analysis.

Finally, regarding the assessment of the risk of developing contralateral breast cancer (CBC), it would have been useful to carry out a comparative analysis using other risk score models such as BRCA-Crisk [38].

We are working to select and enroll new BC families to expand our cases and confirm these preliminary findings. In addition, we will be refining risk stratification and implementing risk scores using different models.

## 5. Conclusions

By integrating PRS with family history, lifestyle factors, imaging risk factors, and carrier status information, healthcare providers and genetic counselors can more accurately estimate breast cancer risks and customize screening and management strategies for families with pathogenic variants in breast cancer-related genes. By identifying high-risk individuals more accurately, healthcare providers can tailor screening and preventive measures, ultimately aiming to reduce breast cancer incidence and improve outcomes for affected families.

## Figures and Tables

**Table 1:** Main characteristics of the 4 families enrolled in the study.

	Subjects (*n*)	Age (Mean ± SD)	Breast Cancer	PV/LPV Carriers (*n*)	Gene	Variant	Pathogenicity Class
Family 1	6	51.5 ± 25.21	Yes: 2 No: 4	2	*BRCA1*	NM_007294.3 c.4117G>T, p.(Glu1373*)	Pathogenic
Family 2	6	44.5 ± 13.30	Yes: 1 No: 5	3	*BRCA2*	NM_000059.4 c.7680dup, p.(Gln2561Serfs*5)	Pathogenic
Family 3	3	49.66 ± 18	Yes: 2 No: 1	3	*PALB2*	NM_024675.4 c.2351_2352delinsT, p.(Lys784Ilefs*67)	Pathogenic
Family 4	3	54.66 ± 11.93	Yes: 1 No: 2	3	*ATM*	NM_000051.4 c.7515+1G>C, p.?	Likely pathogenic

SD, standard deviation; PV, pathogenic variant; LPV, likely pathogenic variant; *, premature termination codon.

**Table 2:** Statistic description of Allelica 577k and 313 SNP PRS models.

	Allelica 577k PRS	313 SNP PRS
Area Under the Receiver Operator Curve	0.71 (95%CI 0.698–0.717)	0.68 (95%CI 0.669–0.688)
Odds ratio per standard deviation	1.81 (95%CI 1.78–1.84)	1.56 (95%CI 1.53–1.58)

CI, confidence interval.

## Data Availability

The original contributions presented in the study are included in the article, further inquiries can be directed to the corresponding author/s.
